# René Dubos, the Autochthonous Flora, and the Discovery of the Microbiome

**DOI:** 10.1007/s10739-022-09692-7

**Published:** 2022-11-08

**Authors:** Nicolas Rasmussen

**Affiliations:** School of Humanities, University of New South Wales, NSW 2052, Sydney, Australia

**Keywords:** René Dubos, Ecology, Flora, Holobiont, Microbiome, Symbiosis

## Abstract

Now characterised by high-throughput sequencing methods that enable the study of microbes without lab culture, the human “microbiome” (the microbial flora of the body) is said to have revolutionary implications for biology and medicine. According to many experts, we must now understand ourselves as “holobionts” like lichen or coral, multispecies superorganisms that consist of animal and symbiotic microbes in combination, because normal physiological function depends on them. Here I explore the 1960s research of biologist René Dubos, a forerunner figure mentioned in some historical accounts of the microbiome, and argue that he arrived at the superorganism concept 40 years before the Human Microbiome Project. This raises the question of why his contribution was not hailed as revolutionary at the time and why Dubos is not remembered for it.

Among recent developments in the life sciences, nothing surpasses the *microbiome* in impact.^1^ Thanks partly to advances in DNA sequencing technology (metagenomics), it has become possible to characterize even those populations of microbes (microbiomes) that cannot be cultured in the lab, including those inhabiting the digestive system and other parts of animals. The US National Institutes of Health conducted a Human Microbiome Project (HMP) from 2007 to 2016 to study the microbiota of the nose, mouth, skin, gut, and urogenital tract of 300 healthy individuals, and it still continues in a second phase tracking changes in the microbial flora associated with certain diseases (US National Institutes of Health n.d.). There are three key changes in thinking associated with the advent of the microbiome. First, the microbe species of the gut and other niches are viewed as an interacting and often self-reinforcing ecological community, metabolically engaged with one another. Second, particularly in the gut, these microbe communities are not only engaged metabolically with the host in terms of digestive processes, but the host responds to their activity both through passive uptake of microbial metabolites and through dedicated signaling pathways—a symbiosis (or dysbiosis) affecting metabolism, immunity, neurological function, and other key physiological processes (Shen et al. 2013; Lederberg et al. 2001; Rizzetto et al. 2018; Barber et al. 2021). Third, it is productive to regard the animal body, together with its co-adapted microbes, as a single organism and the symbiotic flora as organs helping the animal respond to the environment. Essentially, we are superorganisms or *holobionts* (that is, multispecies organisms, like lichens and corals) (Rosenberg and Zilber-Rosenberg 2014; Bordenstein and Theiss 2015; Faure et al. 2018; Simon et al. 2019).^2^ The implications of this new conception for medicine are as great as those for biology; for instance, the microbiota are open to therapeutic intervention by seeding the gut with alternative microbe assemblages, leading to physiological impacts.

A particular history typically accompanies declarations of the new microbiome age. For more than a century (so it goes), there was always some evidence of a constructive role for microbes in health, but this was overlooked due to the dominance of medical bacteriology with its focus on pathogens. But, thanks to high-throughput sequencing revealing the magnitude and complexity of our microflora, a paradigm shift occurred in the 2000s, as microbes were reimagined as indispensable elements of the healthy body (Davies 2001; Relman and Falkow 2001; Juengst 2009; Dupré and O’Malley 2009; Rosenberg and Zilber-Rosenberg 2014; Sangodeyi 2014). In the standard history told by scientists, Russian soil microbiologist Sergei Winogradsky (1856–1953) often features as an early, isolated visionary due to his efforts to cultivate microbes under more natural conditions and in association with other microbes whose metabolic interactions he studied (Rosenberg and Zilber-Rosenberg 2014, chap. 1; Yong 2016, chap. 2). In this he ran counter to the hegemonic German tradition of medical bacteriology, which demanded isolation of microbes in pure culture as a prerequisite to their scientific understanding (a demand, Winogradsky pointed out, that precluded the study of microbe-microbe interaction). As historical work by Lloyd Ackert and others has shown, Winogradsky’s perspective was distinctively holistic, conceiving of the soil and the Earth itself “as a collective entity that possess(es) the characteristic functions of a living organism,” the microbes within it effectively constituting a superorganism that maintains nutrient cycling and therefore the “cycle of life” (Zavarzin 1996; Dworkin and Gutnik 2012; Ackert 2007, 2013; Grote 2018).^3^

French-American bacteriologist René Dubos is also occasionally identified as another forerunner of the microbiome concept. In his prime famous as a pioneer of antibiotic research, and now better remembered for his slogan “think globally, act locally” and other contributions to the environmentalist movement of the 1970s in his retirement years (Gianinazzi 2018), Dubos’s science has recently come to the attention of a few historians for re-evaluation. Focusing on his middle career, Anderson (2004) has cited Dubos as a contributor to modern-day disease ecology history, emphasizing factors of the physical environment rather than biological interactions in an almost Hippocratic manner. Honigsbaum (2017) has questioned the depth of Dubos’s ecological perspective on microbes, suggesting it only emerged mid-career in the context of research on latent infections (especially tuberculosis) and the factors that trigger their activation to cause sickness. In a recent thesis, Sangodeyi (2014) has drawn attention to the work Dubos did in his late research career on the indigenous flora of the gut, arguing that Dubos consciously chose this line of research as a weapon to reform biomedical thinking he regarded as too reductionistic. In this she echoes a thesis written before the microbiome era, where Cooper (1998) argued that Dubos chose the topic of tuberculosis infection and latency in his middle career deliberately as a way to disrupt the dominant reductionism of biomedicine with an ecological perspective on disease. According to Sangodeyi’s analysis, the key distinction between Dubos’s view of the microbiota and the new post-HMP view is that where Dubos saw microbes as part of the biological environment to which the body adapts, biologists now see the microbiota and the animal body as a single organism (Sangodeyi 2014, p. 236).

In this essay I explore Dubos’s thinking about microbial ecology and microbe-host relations, attending especially to his 1960s work on the gut microbiota, which has been little discussed by historical work other than Sangodeyi’s. I will stress the importance of a research technology used by Dubos in this endeavor—the germ-free mouse—together with the field of gnotobiology from which it came. Further, I will stress that, as Dubos himself recollected, his entire research career echoed the ecological ideas of Winogradsky, particularly those that construed the soil microbiota as collectively representing a global digestive organ. Showing how Dubos built on this concept right through his 1960s research on the gut microbiota, clearly articulated as coevolved symbionts and a fully integrated functional part of the body-as-superorganism, I cast doubt on any sharp distinction between his understanding and current visions of the microbiome—and therefore the idea of a recent, seismic intellectual shift within biology about the microbiota. Along the way, I contest the suggestion that Dubos came to think of microbes ecologically only in his mid-career work on tuberculosis, even if explicit ecological terminology in his publications only dates to 1948.^4^ There can be little doubt that Dubos was evoking Winogradsky in his 1940 Harvey lecture when he said “If organic matter does not accumulate in nature, it is because countless species of microorganisms hydrolyze it, oxidize it and eventually break it down to carbon dioxide, ammonia, water, and mineral salts.” And that, in nature, each “microbial species is adapted to the performance of a limited, well-defined biochemical task” so that nutrient cycles flow continually and efficiently (Dubos 1941, p. 406). His research career to that point had been guided by this holistic ecological perspective, as would be his mid-career work on tuberculosis, as Cooper convincingly showed in 1998, and so, I argue, would the rest of it. After showing that Dubos advanced the superorganism concept of the gut microbiota in the 1960s and was recognized for having done so by his biologist peers in the 1970s, I explore the reasons why he has not been widely remembered for this contribution by scientists or the general public, now that the concept’s time has come. Since Dubos left behind few archival records, this essay is centered on the contents and reception of his published work.

## Dubos’s Early Career

In late nineteenth and early twentieth century medical bacteriology, as well as in the popular imagination, the general view was that the microbes in the human gut, thanks to the rich diets and artificial conditions of civilization, took a toxic toll on health—so much so that colonic irrigation was a very popular therapy and some experts seriously recommended removal of the colon entirely (Whorton 2000). However, there were dissenting voices. Louis Pasteur (1822–1895) once speculated that some microbes might play constructive digestive roles and even that animals might not be able to live without their gut flora (Pasteur 1885). His colleague Elie Metchnikoff (1845-1916) became famous for his idea that *Lactobacillus* from yogurt could alter the ecology of the gut so as to displace the toxic species. American bacteriologists Arthur I. Kendall (1877-1959) and Leo Rettger (1874-1954) took up the idea that there was a normal, healthy gut flora that, with the right diet, could defend the body and promote health by outcompeting the pathogenic and toxogenic species (Podolsky 1998; Sangodeyi 2014, chap. 1).While these concepts of the gut flora were ecological in the sense that particular species in the gut were viewed as competing with others and as promoting the better or worse digestion of the host, they did not include the idea of symbiosis. Rather, the notion was that good commensal bacteria should be encouraged to suppress bad ones. The commensals were essentially nontoxic and neutral, perhaps well adapted to the body and representative of the environment in which humans evolved, but beneficial only in comparison to bad microbes. As advertising for yogurt in the 1920s claimed, for example, the product could restore the bacteria “nature intended” (Sangodeyi 2014, p. 74). More sophisticated notions of ecology could be found in agricultural microbiology, as Winogradsky’s example has already illustrated. It would take 1950s research with germ-free animals to substantiate Pasteur’s suspicion that some gut bacteria performed specific digestive functions that benefit humans (Mickelsen 1956; Gustafsson 1959). Like Pasteur and Winogradsky, Dubos’s bacteriological research began in an agricultural rather than medical context.

According to Dubos and his close associates, he first became inspired to study microbiology when, as a young agronomy graduate working for a League of Nations agricultural agency in Rome in the mid-1920s and editing a journal on agricultural science, he read an article by Winogradsky emphasizing the need to study soil microbes in their natural environment in order to learn about the interactions between them (Hirsch and Moberg 1989).The soil science course Dubos had taken at the *Institut national agronomique* in Paris stressed Winogradsky’s perspective, but there is no evidence it made an impression on him (Cooper 1998, p. 34). Encouraged by an American professor associated with his agency, he took a course in bacteriology and prepared to move to the United States to pursue a higher degree in the field. The same professor introduced him to Rutgers soil scientist Jacob Lipman in 1924 at a conference in Rome; Lipman suggested Dubos should come to the New Jersey experiment station that he headed. Dubos also met Rutgers bacteriologist Selman Waksman (1888-1973), who was passing through Rome on a European tour. On that tour Waksman met three times with Winogradsky, whom he admired so much he would write his biography (Hirsch and Moberg 1989; Hotchkiss 2003; Waksman 1946, 1953). This connection was reinforced when Dubos found himself on the same ocean liner as Waksman heading to the US. By the time they arrived in New York, Dubos had been invited to undertake his doctoral work with Waksman at the New Jersey Agricultural Experiment Station and Rutgers (Hirsch and Moberg 1989). Winogradskian thinking and method would permeate Dubos’s doctoral work with Waksman and his subsequent work on antibacterial agents from soil microbes.

In Waksman’s lab, Dubos pursued a thesis project on the identification and isolation of soil microbes that decompose cellulose, which he undertook by developing a defined mineral medium with cellulose as the only source of carbon (energy). This project derived directly from the nutrient cycle work of Winogradsky, with whom Waksman corresponded regularly while supervising Dubos. They largely discussed the importance of studying soil microbes in ways that acknowledged their synergies and antagonisms—and that employed Winogradsky’s enrichment culture techniques, which Dubos was using (Winogradsky 1927; Dubos 1928; see also Cooper 1998, Chap. 2). (Enrichment culture is the method of encouraging or isolating one microbe species in an initially mixed culture by imposing nutritional or environmental conditions that particularly favor its growth).

Toward the end of his doctoral studies, Dubos paid a visit to the Rockefeller Institute in New York, where he met fellow Frenchman Alexis Carrel (1873-1944) and, more consequentially, bacteriologist Oswald Avery (1877-1955). Although there are conflicting accounts of who initiated this second introduction, all agree that Avery, who was working on the polysaccharide capsule of virulent strains of pneumococcus (*Streptococcus pneumoniae*), was impressed by the possibility that Dubos might find soil bacteria that produced enzymes to break down the capsules in the same way as he had with cellulose (also a polysaccharide).^5^ In late 1927, Dubos began working with Avery at the Rockefeller Institute and in 1929, after more than a year of feeding soil sample cultures nothing but pneumococcus polysaccharide, finally found a bacterium that produced the desired enzyme (McCarty 1985, pp. 70–71; Waksman 1960, p. 504; Cooper 1998, p. 111).^6^ With this discovery, Avery proved that the virulent strain owed its lethality to the capsule and that, under certain conditions, injections of the enzyme could even cure infected mice (Avery and Dubos 1930; Dubos and Avery 1931; Avery, Oswald, and Dubos 1931). In follow-up studies in the early 1930s, Dubos explored the way factors such as chills and poor nutrition could affect the ability of animals to defeat pneumococcus infections after treatment with the enzyme, which, as Cooper pointed out, clearly foreshadowed the topic that would preoccupy him from the early 1940s to late 1950s—tuberculosis latency—and adds weight to her argument of ecologically-informed continuity between Dubos’s early- and mid-career research (Goodner and Dubos 1932; Cooper 1998, pp. 120–122).

While Waksman was not shy about stressing his own role in Dubos’s successes—for instance, emphasizing that he supplied Dubos the cranberry bog soil that yielded Avery’s enzyme—he credited this work by Dubos as showing the way forward in antibiotic discovery that would lead to his own Nobel Prize in 1952 for the discovery of streptomycin. In 1960, Waksman wrote:

Although the method of enriching soil with particular nutrients, followed by the isolation of organisms capable of attacking such nutrients, was well known in the study of soil microbes (since the work of S. N. Winogradsky and M. W. Beijerinck), this development was rather unique in medical microbiology. Dubos deserves the credit for applying this method to the isolation from soil of organisms capable of attacking pathogenic bacteria or their cellular constituents. (Waksman 1960, p. 112)

Immediately after his pneumococcus success, however, Dubos did not follow his Winogradskian conviction that “soil enrichment [w]as a technique for recovering microbes that could do almost anything” in the direction of new antimicrobial agents (Hirsch and Moberg 1989, p. 137). Rather, he used these techniques to discover new enzymes with other medical uses, such as one that specifically broke down creatinine and could be used in a blood test for that substance (Dubos and Miller 1937; Dubos 1941).

By 1937, however, Dubos had turned the enrichment culture method to the discovery of soil bacteria, and the substances they secreted, that could attack bacteria pathogenic to mammals. Using a simple mineral medium and soil samples, much as he had in his search for a cellulose-degrading microbe with Waksman and the pneumococcus capsule-degrading enzyme with Avery, for two years Dubos fed mixed soil bacteria on living *Streptococcus* cells as their only source of carbon. Eventually, he found a culture that destroyed samples of live *Streptococcus*, isolated the bacterium (*Bacillus brevis*) that produced the bacteriocidal agent, and, with Rockefeller Institute chemist Rollin Hotchkiss, purified its two active ingredients, christened tyrocidin and gramicidin. The first was as poisonous to animal cells as to *Streptococcus*, but gramicidin could fight infection in experimental animals and found clinical use as a topical antibiotic (Dubos 1939a, 1939b; Dubos and Hotchkiss 1941). This work inspired a new wave of research to develop other antibiotics, such as the work by Howard Florey (1898-1968) and Ernst B. Chain (1906-1979) on penicillin and Waksman’s on streptomycin, already mentioned. It also won Dubos considerable acclaim (Hirsch and Moberg 1989).^7^

## Dubos’s Work in the 1940 and 1950s

As his biographers and close associates have written, Dubos’s research entered its second of three periods in 1942, when he went to Harvard for a two-year sojourn before returning to the Rockefeller Institute as director of his own lab. While at Harvard, he was unable to work on tuberculosis as he planned; instead, he led a classified biological warfare research project on the dysentery-producing bacterium *Shigella* with both offensive and defensive dimensions (Fitzgerald 1993, chap. 5; Cooper 1998, pp. 166–169).^8^ Whereas the first period just described can be viewed as following the classic soil ecology paradigm pioneered by Winogradsky because of its emphasis on the bacterial agents of nutrient cycling and microbe-microbe interactions in mixed culture, as well as its use of enrichment culture method, the second has been called the period of “experimental pathology” by Dubos’s biographers and, equally suitably, the “disease ecology” period by Honigsbaum. During this period, Dubos studied interactions between pathogenic bacteria and their animal hosts, together with factors influencing the host’s ability to suppress disease, although straying from time to time into the effects of other microbes on both pathogen and host resistance. One might view this work as extending the Winogradskian paradigm to animals—the soil in which pathogenic bacteria thrive. Or, in addition, it also could be seen as an application of the insights Dubos had gained from his plant pathology instructors at Rutgers, Conrad Haenseler and Howard Sprague, who emphasized how environmental conditions (temperature, light, nutrition, soil pH) could prevent or cure infectious disease (Cooper 1998, pp. 67–68). Much of Dubos’s mid-career research focussed on tuberculosis—in particular, on the factors that determine whether a host can suppress TB infection to latency or instead fails so that the mycobacterium escapes control to produce florid disease. Honigsbaum (2017) plausibly suggested that his keen interest in this topic was driven by the death of his first wife, whose latent TB infection’s resurgence he attributed to her mental stress.

Since the period of Dubos’s research from 1944 to the late 1950s has been ably analyzed by others, notably by Cooper (1998) and Honigsbaum (2017), I will describe it only in so far as is necessary as to connect it with the subsequent period in which Dubos explored the gut flora. In this respect, the most interesting work Dubos did in the middle period is that dealing with the influences of a second microbe on tuberculosis infection and host defense, and with the ways in which environmental factors *within* the body related to host-microbe interactions. As to microbe-microbe interactions within the host, Dubos’s published work from the late 1940s shows only a few signs of interest. For example, in one paper Dubos and collaborators showed that concurrent lung infection with a virus, at levels insufficient to produce disease on its own, greatly increased the rate at which mice injected with TB bacilli developed gross lung lesions (Volkert et al. 1947). As with some similar findings, in his interpretation Dubos refrained from stating whether the effect was due to a co-adapted synergy among microbes or instead due to the generic insult of a second infection (Dubos and Schaedler 1956; Schaedler and Dubos 1957).

Most of the work Dubos did on the factors affecting TB and other infection latency had little to do with either microbe-microbe interaction or microbe niche within the body. Rather, it expanded on the insight that infection by a pathogen does not lead automatically to disease (a blow against Germanic reductionism, as Cooper argued (1998). Infectious diseases have multiple causes, including both internal and external “environments” as well as pathogens (see Dubos 1954). Thus, any generic insult to the host can weaken its defences so that an infection that would in better circumstances be cleared or suppressed will instead take hold and produce lethal disease. Factors that Dubos demonstrated as resistance-weakening include deficient diet, transitory fasting, chilling, and accelerated metabolic rate with drugs. Partly to distinguish the effects of these insults and stresses on bacterial multiplication rates, as distinct from host sensitivity to bacterial toxins, he began to work with faster-growing bacteria that could also opportunistically cause disease, such as *Staphlococcus aureus* and *Klebsiella pneumoniae*. In general, insults did accelerate bacterial growth and some also lowered toxin resistance, measured by injection with killed bacteria. Thus, the body seemed to stave off disease by actively creating an unfavorable environment for the growth of bacteria that found their way into tissues—defensive processes that stressors could impair (Dubos, Smith, and Schaedler 1955; Smith and Dubos 1956; Dubos and Schaedler 1958).

Dubos also attempted to explore the particular processes that the body uses to suppress the growth of invading pathogens. That is, during this period Dubos was not just interested in the impact of the outer world experienced by the host upon microbes within it, but also the impacts of change in a host’s interior—the immediate environment of the microbes. As Hongisbaum and Anderson observed, Dubos differed from most contemporary microbiologists in emphasizing physical and chemical factors as determinants of infection control as opposed to specific immunological defense mechanisms. However, this interest in physical and chemical explanations reflects not (or not just) a deficiency in Dubos’s immunological sophistication but rather an extension of the soil microbiologist’s perspective to the body’s interior. Dubos went as far as contemporary methods allowed to characterize the physical and chemical properties of the immediate tissue environment of bacteria affecting their invasiveness. He also explored how these internal environment factors are manipulated by the host and distinguished local changes in the internal environment from the overall defense-weakening effects of generic insults, which would include lowered specific immunological activity. For example, he found that survival of TB-infected mice was improved by diets enriched in lactic acid and worsened in mice subjected to weekly 30-hour fasting, which induces ketosis. Yet mice fed the same diet in restricted quantities such that overall growth rates were lower than the fasted mice were not worsened (despite their semi-starvation). This finding was consistent with in vitro studies showing that growth of cultured TB bacilli was retarded by lactate and enhanced by ketones in the medium, and thus consistent with the hypothesis that lactic acid production at the site of infection was a mechanism of infection suppression (Dubos 1955). Similarly, Dubos found that TB bacilli cultured in low oxygen lost their virulence and even died, especially when exposed to lactic acid, suggesting that the low oxygen environment created by the body when it encased the bacilli in granulomas was a key defense mechanism against the disease (Dubos 1953).

To Dubos, the mouse was not just a living test tube to cultivate bacteria nor even a complex terrain containing multiple niches for microbes. It was an active partner-opponent to the microbes, co-adapted to the microbial world that represented its biological environment, just as much as the body was the environment to the microbes within. Still, in all his mid-career research on how animals bring potentially dangerous microbes “under metabolic control” so that the host may “live at peace” with them, there is no hint that he believed that the microbes within could actually be a cause of good health (Dubos 1955, p. 1479; Dubos 1958). This concept emerged in his late research career.

## From Disease Resistance to Gut Flora, 1958–1970

Some of Dubos’s mid-1950s experiments into dietary deprivation had turned up the curious fact that certain types of diet, even though adequate in nutritional content, impaired the ability of mice to survive bacterial infection. For example, mice fed on the milk protein casein fared worse when injected with bacterial toxins than those fed on normal mouse chow, even when the casein was supplemented with amino acids it was low in. It seemed to Dubos that an optimum “balance” of nutrients in the diet was necessary for full resistance to infectious disease (Dubos, Smith, and Schaedler 1955). Pursuing this issue, Dubos and his group found that mice fed casein as the protein source showed better disease resistance, measured by survival time after injection with several kinds of live pathogens, than mice fed even less suitable diets containing the same amount of protein from corn, wheat gluten, or soybean. However, a mixture of soybean and rice flour with roughly the same amino acid profile as casein yielded similar resistance, fitting with the balance idea. Moreover, the group found that the quantity of casein in the diet made a great difference: the higher casein diets yielded better survival than lower, even when the lower levels had enough protein to support the same rate of growth in the young mice. That is, aspects of disease resistance depend on diet in ways other than nutritional adequacy and other than balance too (since high and low casein diets would offer the same amino acid ratios) (Dubos and Schaedler 1958). This finding strengthened the view that, beyond mere sufficiency to support growth, the quality or kind of diet had an impact on disease resistance. How and why did food quality matter? By 1958, Dubos must have begun to suspect not so much the exact chemical content of the diet as a crucial factor but rather its effect on the bacterial populations inhabiting the gut of his mice. After all, the food in the gut was the soil in which these diverse microbes lived, and as Dubos well knew from his early-career research, in different soils bacteria grow and behave differently. That was the year he sought new experimental mice that would allow him some control over the gut flora.^9^

Such creatures did exist, produced in the strange domain of “gnotobiology” where biologists, some of whom were funded under secret germ warfare research programs, nurtured lines of animals born from cesarean section and handfed on sterilized milk through gloveboxes in germ-free isolators behind airlocks (Reyniers 1959; Kirk 2012). They lived without microbes, but they were not normal and were not entirely healthy. In late 1958, in collaboration with the Rockefeller Institute’s J. B. Nelson, who had earlier established a colony of rats from animals bred by the doyen of American gnotobiology, Notre Dame’s James Reyniers, Dubos, using similar techniques, established a mouse colony with a simple gut flora and free from specific known pathogens that he called NCS mice (Dubos and Schaedler 1960; Nelson and Collins 1961; Nelson 1951).^10^ In some ways these NCS mice seemed healthier than both germfree animals and the conventional mice from which they were derived. They grew faster than their germy counterparts and far faster on inferior diets like those based on gluten. They were more fertile, and not only due to lower infant mortality. However, they succumbed to some bacterial infections much more easily since their immune systems had no prior exposure to pathogenic microbes. Perhaps more surprisingly, the NCS mice proved far more resistant to injections of bacterial toxins. But most unexpectedly, Dubos found that introducing the commonplace, occasionally pathogenic gut bacterium *Escherichia coli* to NCS mice, where it was initially absent, made them just like conventional mice in terms of growth rates, response to deficient diets, resistance to infection, and resistance to toxins. And *E. coli* was usually regarded as a normal part of the gut flora of mice as well as humans. As Dubos summed it up, the implications were profound: “It is clear that many characteristics assumed to be inherent in an individual can in reality be determined by the microbial flora of the intestinal tract” (Dubos and Schaedler 1960, p. 416).

Dubos and his group began looking more closely at the bacteria in the guts of his NCS mice and their conventional counterparts to characterize the flora and to investigate which microbes in particular were responsible for what physiological effects. Comparing his NCS colony to six other mouse colonies of similar genetic derivation, he found that while all had large quantities of lactobacilli in their feces, the NCS mice had an order of magnitude more and, uniquely, had a particular type characterized by a “rhizoid” colony form. The NCS mice, on the other hand, carried four orders of magnitude fewer enterococci and Gram-negative bacilli—and no micrococci, *Proteus*, *Pseudomonas*, or *E. coli*. After treatment with antibiotics or even after mild stress like handling or being caged alone, the NCS mice tended to suffer an increase in Gram-negative bacilli, a decline in lactobacillus, and an increased susceptibility to endotoxins. The microbial community conducive to health in these animals had been displaced through these environmental stressors by a less friendly but commonplace gut population (Schaedler and Dubos 1962). In another study looking only at NCS mice on different diets, Dubos found that poor diets such as those based on gluten or casein caused a decrease especially in the rhizoid lactobacilli, and these same diets also increased susceptibility, both to infection and, to a limited extent, endotoxins. So, it seemed that the better health of the NCS mice that Dubos had previously discovered only manifested when they bore a gut population characterized by abundant rhizoid lactobacilli, although even the low-rhizoid NCS mice were still more resistant to endotoxins than those colonized by *E. coli* (Dubos and Schaedler 1962a). Thus far he had essentially vindicated predecessors like Metchnikoff and Rettger, who argued that the right gut flora promoted health by excluding more deleterious microbes.

By 1962, Dubos felt he had convincingly shown that mice with gut flora typical of his NCS mice on a proper diet—high in *Lactobacillus* species, low in enterococci and coliform bacteria, and lacking in *Proteus* and *Pseudomonas* species—reproduced more efficiently, grew faster on suboptimal diets, and resisted bacterial endotoxins much better. Also, this flora was self-sustaining on the right diet, even when his NCS mice were exposed to the less healthy gut flora of conventional lab mice. But he was not yet prepared to attribute their better health to the abundance of lactobacilli and other NCS gut flora per se, especially regarding infection resistance; the flora correlated with host physiology, but he felt both might share a common unknown cause (Dubos and Schaedler 1962b). To go further in saying that physiological effects were actually induced or caused by a particular flora, he would need to vary the microbial population of the gut at will in otherwise identical mice. This was the way that the gut flora in rats had recently been proven by others to serve important nutritional functions (Gustaffson 1959; Daft et al. 1963).

And so, Dubos turned again to gnotobiology, sourcing fully germ-free mice from the commercial Carworth Farm animal breeders nearby. He also began to put great effort into identifying bacteria that were strict anaerobes and otherwise hard to culture, hence difficult to characterize (Schaedler, Dubos, and Costello 1965). With new methods and media for culturing strictly anaerobic bacteria, he was able to show that the anaerobes were far more abundant in feces than previously suspected—a million times more abundant in his healthy NCS mice than the enterobacteriaceae (like *E. coli*), the family traditionally thought of as typifying the normal flora. He found that the unhealthy casein diet decreased numbers of the anaerobic group N lactic streptococci and lactobacilli but increased the Bacteroides species. Further, with the new methods, he recovered the flora not only from feces but also from the contents and tissue of the different segments of the digestive tract. He found that in the cecum (upper large intestine) of his NCS mice there were as many group N streptococci as there were lactobacilli, and also plentiful Bacteroides as well as much less plentiful enterobacteriaceae and enterococci, all consistent with his earlier cultures from feces. Looking at the stomach and small intestine, organs previously thought to harbor few bacteria, he found them teeming with anaerobes, mainly lactobacilli and group N streptococci again, and no Bacteroides species or enterobacteriaceae. Strikingly, he found that in all these parts, there were larger numbers of lactobacilli and group Ns actually living in the walls of the organs (recovered by washing and then grinding the tissue) than in the contents moving through them. These bacteria apparently colonized the surfaces of the animal tissues (Schaedler, Dubos, and Costello 1965; Dubos, Schaedler, Costello, and Hoet 1965).

Using animals maintained in fully germ-free conditions, with collaborator Russel Schaedler he followed this colonization process by separately inoculating germ-free mouse pups with a culture of one strain of Bacteroides from his NCS mice, one strain of group N *Streptococcus*, and a mixture of a non-rhizoid and rhizoid strain of *Lactobacillus*. The lactobacilli and group Ns quickly became abundant in the walls of all three parts of the gut, while the Bacteroides colonized only the large intestine—and, in the process, corrected one of the most dramatic abnormalities of germ-free mice. Among their other differences from normal mice, including such features as immature immune tissues and altered metabolism, the cecum of germ-free mice is incompletely differentiated and oversized, up to one third of the animal’s weight. But shortly after colonization with Bacteroides in particular, the group found that it returned to a much more normal form and size (Schaedler, Dubos, and Costello 1965; Gordon and Pesti 1971).

Already by 1964, both his experiments with NCS animals and this new work with true germ-free mice had transformed Dubos’s perspective on the gut flora in a way that can be viewed as marking the advent of today’s concept of the microbiome. In a medical conference talk that year entitled “The Digestive Tract as an Ecosystem,” Dubos explained that these studies had brought him to distinguish between the “so-called normal” flora—the coliform bacilli, enterococci (whose names bespeak their place in the medical imagination) and the clostridia—on the one hand, and, on the other, what he dubbed the “autochthonous flora” (the very term Winogradksy used for the native flora of a given soil) (Dubos and Schaedler 1964; Waksman 1946). The “so-called normal” flora loomed large in medical microbiology, as these microbes were pathogens even though they could often be found in healthy people, a fact that grounded the old concept that typical gut bacteria were pathogens in waiting, kept in check except when the host was weakened, much like the latent tuberculosis germs he had long been studying. Dubos echoed the common wisdom about these microbes, calling them “accidental invaders … organisms which are ubiquitous in the environment, and which can gain a foothold in the body … [not] essential to the host’s welfare, and indeed probably somewhat detrimental under many circumstances.” But the autochthonous flora were, in contrast, “intimately associated with their host in a symbiotic relationship,” not only helping the host extract nutrients from food but also, through their varying metabolic products, modulating the host’s biology to adapt to its external environment. These of course were the lactobacilli, anaerobic (group N) streptococci, the Bacteroides group “and certainly other anaerobic species not yet identified.” Each of these symbiotic microbe types were at least 10,000 times more common in the healthy (NCS) mouse gut than the coliforms and enterococci that constituted the “normal flora” of medical bacteriology and, as noted, they occupied distinct niches within the living tissue. In sum, the autochthonous gut bacteria together with their mammal host, living in symbiosis, constituted a “true ecosystem” that determined health (Dubos and Schaedler 1964).

Over the next few years Dubos and collaborators Schaedler and Dwayne Savage elaborated this view of body-as-ecosystem. For example, they designated an unculturable fusiform anaerobe (now known as *Faecalibacterium*) the dominant member of the autochthonous lower intestine flora, finding that in at least some cases microbes occupied very particular anatomic niches “in almost pure culture.” The animal body was occupied by many species of co-adapted, symbiotic microbes, each in a particular place and in contact with particular tissues, without which the body could not function normally (Savage and Dubos 1967, p. 1811; Savage, Dubos, and Schaedler 1968). This is the superorganism concept. Thus, the autochthonous gut flora were not merely a living element of the environment located inside the body, as Sangodeyi argued. Rather, they were for Dubos biologically integral to the body, helping regulate its function appropriately. By the 1970s Dubos was advocating that every infant be subject to a “controlled and systematic colonization by … the autochthonous flora [because] these species are truly symbiotic with man and are indeed essential for the normal development of his tissues” (Dubos 1975, p. 18).

## The Professional Reception of Dubos’s Microflora Research

If Dubos articulated the superorganism concept with respect to the gut microbiota in the 1960s, how did his scientific contemporaries receive this? Biological research into animal microbiota, especially the gut flora, was a large field by the end of the 1960s, and within it the notion that some species of our native flora do us more good than harm was uncontroversial. During the 1970s, the field moved beyond Dubos in methods for studying the flora but he was certainly recognized as a founding figure. At the First International Symposium on the Ecology of the Intestinal Flora in a Changing Environment, where leading gnotobiologist Thomas Luckey convened a Who’s Who of the most active researchers in the field in 1970, Luckey called for a fusion of newer methods of characterizing anaerobic bacteria with the “Dubos school” approach to investigating the way host tissues responded to symbiotic microbes (Luckey 1970, p. 1540). One major bacteriology review from 1971 cited Dubos and collaborators as champions of the true symbiosis idea and as having discovered the way in which the indigenous (“autochthonous” for Dubos) gut flora, particularly Bacteroides species, become established in young animals (Gordon and Pesti 1971). Another prominent review that same year similarly credited the Dubos group as having shown how the indigenous flora become intimately established within tissues of the gut (Gorbach 1971). A 1973 review in the *New England Journal of Medicine* dealing with drug metabolism cited the Dubos group as showing that the gut microflora influence nutrition, metabolism, and disease resistance and as suggesting that “the intestinal microflora might be regarded as an organ of the body” (Goldman 1973). In sum, Dubos said that the autochthonous bacteria were true symbionts and needed to be seen as an active part of the animal or, better for practical and medical purposes, the animal and its autochthonous flora together constituted the organism. His scientific contemporaries heard him.

In research publications about the intestinal microflora of the 1980s, citations to Dubos are rare, which is unsurprising since scientific literature mainly cites journal articles less than ten years old and citations to more recent review articles tend to replace citations to classic original findings (McMahan and McFarland 2021). However, by looking at the publications of thought leaders in gastrointestinal microbiology from the 1970s to the 1990s, one can see that many of the ideas Dubos promoted in the 1960s became increasingly mainstream. To focus on Dubos’s notion of an autochthonous gut flora that is genuinely symbiotic and essential for health (because other aspects of the superorganism concept—the notions of the flora as a metabolic organ and as an ecological community—were also associated with other figures in the field; Savage 2001),^11^was taken as given in the 1970s by some, like M. H. Floch and Sherwood Gorbach (Floch 1974; Bartlett et al. 1978).^12^ Others, like Dirk van der Waaij, appear to have accepted it gradually in the 1980s;^13^while still others, like Tomotari Mitsuoka, do not appear to have accepted the gut flora as symbiotic—as opposed to merely commensal—before the 1990s.^14^ Indeed, during the late 1980s and 1990s, Dubos continued to be cited occasionally in review articles authored by senior members of the gut microflora research community to which he belonged in the 1960s (see van der Waaij 1988). Thus, it would be incorrect to say his contribution was forgotten by them. Rather, if Dubos is not hailed by scientists today as a prophet of the superorganism idea, it seems more likely that newcomers to the field in the genomics era never learned of his contributions—nor, one supposes, of much earlier work in the field.^15^ These molecular geneticists of a new generation, attracted to the gut microbiota by the emerging sequencing-based methods for identifying hard-to-culture microflora, then discovered the superorganism concept as if it were a new idea.

Another factor to consider about the way Dubos has been remembered, both by scientists new to the field and by outsiders, is his construction and commemoration as a historical figure from the 1990s onward. There is a large literature on commemoration and the making of historical memory (Halbwachs 1980; Lowenthal 1985; Nora 1989; Kämmen 1991; for modern biology see Abir-Am 1999), but for present purposes, it suffices to recall that all shared memory is culturally and intellectually situated, and motivated by its makers to shape that situation. The most active contributor to published literature on Dubos, and effectively the custodian of his memory, has been Carol Moberg, Dubos’s assistant at Rockefeller late in his career. She co-authored his official biographical entry for the US National Academy of Sciences, wrote a number of historical reflection pieces about him in scientific publications as well as a full-length biography, and contributed a feature on him in *Scientific American* magazine (Hirsch and Moberg 1989; Moberg 1990; Moberg and Cohn 1991; Moberg 1999; Moberg 2005). In these writings, there is scant mention of Dubos’s work on the gut microflora and his late career research in general. Rather, Moberg’s writings all point out that Dubos was the first to show how antibiotics could be discovered from soil samples and that this work led directly to the Nobel-winning research of Waksman and Chain and Florey. Many pieces highlight Dubos’s discovery that the bacterium that produced his *Pneumococcus* capsule-degrading enzyme did so only when the capsule substrate was present, an example of the *adaptive* or *inducible* enzymes whose study won François Jacob and Jacques Monod their Nobel Prize. They also frequently credit Dubos as one of the first to predict the rapid evolution of antibiotic-resistant pathogens, a problem of current and still mounting importance. The implication is that Dubos was a brilliant thinker who originated ideas that won others Nobel Prizes (in which, perhaps, he should have shared) and, in general, was far ahead of his time.

It would not be highly speculative to suppose a motivation on Moberg’s part to win Dubos greater posthumous credit. The attendant focus on science considered important, or vindicated, at the time of writing in the 1990s and early 2000s—molecular genetics, antibiotic discovery, antibiotic resistance—would conversely generate neglect of Dubos’s work that did not resonate with then-current trends. This fits with the otherwise favorable review of Moberg’s monograph, *René Dubos, Friend of the Good Earth* by Dubos’s onetime collaborator Schaedler, who complained that she gave very short shrift to his late career research in general, especially the gut flora research, and even reproduced the disparaging comment of another scientist that it was “not worth a hill of beans” (Schaedler 2006; Moberg 2005, p. 126). That is, for Schaedler, Moburg had under-emphasized and under-rated exactly that part of Dubos’s work which today could be seen as foundational for microbiome biology. Thus, ironically, a credit-seeking motivation on Moberg’s part can explain why Dubos is not credited with the superorganism view of the gut microbiota, because it had not yet achieved currency when most of her relevant work was written. If Moberg’s emphases had been otherwise, the new generation of microbiome sequencers might have honored Dubos more.

## Conclusion: Dubos’s Popular Reception

In closing, we might ask why Dubos was not remembered for his ideas about the symbiotic gut flora and his overarching symbiosis-superorganism vision among the general public, despite his high profile in the 1970s. After all, if he were popularly remembered for promoting these ideas, the scientific community might never have forgotten. Upon his retirement as a Rockefeller laboratory director in 1970, Dubos took up the role of public intellectual afforded by the 1969 Pulitzer Prize he won for his book *So Human an Animal* (Dubos 1968) Even though he had attempted in *So Human an Animal* to balance such images as landing on the moon while knee deep in garbage on Earth with more hopeful messages,^16^ reviewers saw him as “gloomy on environment” (Leonard 1968) and much of the acclaim focussed on Dubos’s account of how badly degraded the Earth had become. In reaction to what he felt was excessive negativity, Dubos thenceforth took on an identity as a “despairing optimist” and “free lance of the environmental movement” that clashed with the mainstream by promoting the idea that humanity could learn to live harmoniously with nature by inhabiting it creatively and even improving it in the manner of the traditional European rural landscape. In the regular columns that he wrote and the many, often large large public addresses—40 on average per year—that he gave over the next decade, he stressed this “optimistic” view of human agency and our place in nature (Moberg 2005, pp. 140–153). And he was popularly remembered for this stance; for example, his *Toronto Star* obituary explained that Dubos believed “Man does not ruin his environment simply by changing it” (Anon 1982).

Thus, as Sangodeyi observed, in all his popular works, from *So Human an Animal* onward, Dubos advanced the central argument that humans adapt to their environments for better or worse, and that since we produce our own environments, we must now make our Earth suitable again for human flourishing. In making this case for environmental activism, he might have discussed our co-adapted microflora as another part of the environment that can degrade or ennoble us, or else as a part of us that helps us adapt to external change. He often brought up examples from biology in his arguments. But surprisingly, given that the subject occupied him professionally for a decade, there is minimal mention of the gut microflora in Dubos’s popular books.^17^ There is no mention at all in *So Human an Animal *(1968), *God Within *(1972b), *Beast or Angel *(1974), or *Wooing of Earth *(1980). In contrast, the first three of these works do mention tuberculosis, as does his final book *Celebrations of Life* (1981). This last book contains an extended discussion of biological symbiosis, which he takes in a Kropotkin-like direction. There he does mention that humans, like cockroaches and cows, require “certain bacteria” for intestinal health, but only devotes three lines to our symbiotic flora as opposed to twenty-five to the symbiosis of lichens (Dubos 1981, pp. 185–188)—a subject in which he was not particularly expert.

Dubos thought carefully about his public messaging and its likely impacts—what he called his “public action.” For example, although often asked by leftists with whom he was sympathetic, he made it “an absolute rule” not to speak about the Vietnam War because whatever he said could be dismissed or “used wrong” because of his French origins (Mitchell 1972, B5). Thus, we may justifiably suppose that he consciously chose to say very little in the public sphere about the symbiotic gut flora that occupied so much of his scientific career and ask why. He could not have imagined that his audiences would not understand or be receptive to a detailed account of how our health depended on the right ecological community in our guts, given widespread consumer receptivity by the 1970s to advertising claims around our “natural, good bacteria” (Roszak 1995; Sangodeyi 2014, chap. 4). To speculate, in the absence of relevant archival information, I would suggest that the reason for Dubos’s neglect of our flora in his popular works is that assigning agency over our health to the microbes in our guts would align him more to the mainstream ecology activists he was trying to distinguish himself from, with their message of human vulnerability and dependency on the Earth’s ecosystems. This would complicate and partly undermine his central message that the choice of world is ours to make. This guess is consistent with an analysis of thirty popular periodical publications either by Dubos or containing an interview that appeared between 1970 and 1980. In these too he never mentions the microbiota. Tellingly, in the few instances that mention symbiosis, instead of the gut flora we might expect by way of illustration, he discusses humanized landscapes—a favorite topic—to demonstrate how positive anthropogenic change can sometimes be when a culture has a healthy relationship with the Earth. Here Dubos implicitly positions humanity as a symbiont in the bowels of the planet (for example, Mitchell 1972, Dubos 1972a). In any case, he consistently took care to focus all agency squarely on humanity, even when he acknowledged that the symbiosis is reciprocal. “Man creates himself,” Dubos liked to say (Dubos 1965b, p. 15), and he would not want to risk even the appearance of contradiction by describing how microbes shape us, where he saw the human future at stake.

## Footnotes

1 The *microbiome* term and concept is often misattributed to Joshua Lederberg. The term is much older and was being used in a manner cognate to its present meaning in the 1980s, although *microbiota* was more common (Prescott 2017). As for the current concept of the microbiome, when Lederberg used the term around 2000 (Lederberg and McCray 2001 is often cited, although the term does not appear there) it had additional connotations due to the currency of the Human Genome Project and technological advances enabling sequencing of environmental DNA as a way to characterise non-culturable microbes. For a review of these techniques circa 2000, see Rappé and Giovannoni (2003).

2 For a critique of the way current holobiont theory melds physiological and evolutionary reasoning, see O’Malley (2017).

3 Winogradsky as cited in Ackert (2007, pp. 109–145).

4 It may well be that in the late 1940s Dubos’s thinking about disease was influenced by contact with the (natural) selectionist views of Burnet in the early 1940s—keeping in mind that as a Frenchman educated in the 1920s, his initial understanding of adaptation may have been as neo-Lamarckian as Darwinian. But *ecological* thinking, concerning the dynamic relations between different organisms and their environment, needs to be distinguished from *evolutionary* thinking, even though they are often conflated and combined by the way evolution by natural selection is the dominant explanation of how ecological relations came to be. On the state of evolutionary theory in the 1920s, see Bowler (1992).

5 Hirsch and Moberg (1989) and Cooper (1998), echoing Dubos’s own recollections, said that he sat next to Avery accidentally at lunch and proposed to Avery that he could easily find a soil microbe to break down pneumococcal capsules. Avery’s associate Maclyn McCarty said it was Avery who first had the idea that a Waksman student might find his desired enzyme (McCarty 1985, pp. 68–70), whereas Waksman claimed the credit for initiating the meeting himself (Waksman 1960).

6 Cooper (1998), following Dubos’s oral history recollections, stated that Dubos spent at least his first year with Avery learning the basics of medical microbiology and that it took “only a few months” (p. 111) to find a capsule-degrading microbe once he actually tried soil sample enrichment cultures. I am inclined to accept McCarty’s account of more than a year’s isolation effort since it acknowledges that Dubos initially undertook other activities.

7 Cooper (1998, chap. 4) argues that Dubos’s search for antibiotics was motivated by concern about the toxicity of the newly introduced sulfa drugs and the idea that biological control might provide a gentler therapeutic.

8 Three particular publications suggest the nature of his wartime research on Shigella as a biological weapon: Dubos, Hoberman, and Pierce (1942); Dubos et al. (1943); Dubos and Geiger (1946).

9 J. B. Nelson started the NCS mouse colony for Dubos in October 1958 according to Dubos and Schaedler (1960). In a talk given in May 1962, Dubos spoke “of four year’s personal experience” with his disease-free mouse colony (Dubos 1962).

10 Dubos was a member of the National Academy of Sciences biological warfare committee in George Merck’s War Research Service, as was Reyniers. Reyniers collaborated intensively with several of Dubos’s fellow research project leaders, including Karl Meyer in Berkeley, with whom Dubos had earlier worked closely (Meyer et al. 1937). In any case, the members of the committee were aware of one another’s projects: See “DEF Committee: Present Status of Research Projects Initiated by War Research Service,“ 12 Oct. 1944, Botany Department Papers, box 7, folder 2, University of Chicago Archives. See also Fitzgerald (1993, chaps. 4–5).

11 The notion of the flora as a metabolic organ and that it constituted an ecological community was associated with the group of Bengt Gustaffson, on one hand, and the groups of Thomas Luckey and H. Williams Smith, on the other, as well as Dubos (for example, Smith and Crabb 1961; Luckey 1970; Gustafsson 1982; see Savage 2001).

12 Gorbach (1971) distinguishes *commensal* from *parasitic* gut microbes, and also speaks of “commensals and symbions” (see Bartlett et al. 1978).

13 Compare van der Waaij and Berghuis-de Vries (1974) with van der Waaij (1987). The latter treats the “indigenous” anaerobic flora as a barrier or “living wall paper” in the gut, not just as a community that resists colonisation by invaders.

14 Mitsuoka recently seems to attribute the idea of a symbiotic, beneficial flora to himself, not Dubos (Mitsuoka 2014); however, the piece to which he refers (Mitsuoka 1992) only discusses symbiosis *among* bacteria in the normal flora, not with the host.

15 A systematic citation analysis would be needed to establish it convincingly, but this impression is borne out by a casual survey of 10 highly-cited review articles on the gut microbiome published between 2005 and 2010 as identified by Google Scholar. None cited Dubos, nor most of the other early researchers identified as “pioneers” by Savage (2001), while four cited the piece by Lederberg and McCray (2001) discussed in footnote 1, above.

16 Dubos actually drew this memorable image from a speech by US Secretary of the Interior Morris Udall, who in turn credited it to Cleveland, Ohio’s mayor, but it is likely that most readers heard it first from Dubos (1968, p. 231).

17 To be sure, a chapter is devoted to the “indigenous microbiota” in his 1965 *Man Adapting*, derived from his Silliman Lectures at Yale and largely based on his own research, in which he described our autochthonous flora as genuine symbionts on which we are dependent, much like the algae are to fungi in lichen. But this work is aimed mainly at an academic audience, was written before his role as environmental activist, and lacks any overt political agenda (Dubos 1965a, pp. 89–92, 112).
